# Toward a Financially Sustainable Healthcare System in Saudi Arabia

**DOI:** 10.7759/cureus.46781

**Published:** 2023-10-10

**Authors:** Fayez Almodhen, Wael M Moneir

**Affiliations:** 1 Pediatric Urology, Surgery Department, King Abdullah Specialist Children's Hospital, King Abdulaziz Medical City, National Guard Health Affairs, Riyadh, SAU; 2 Pediatric Urology, King Abdulaziz Medical City, Riyadh, SAU

**Keywords:** sustainability model, vision 2030, saudi arabia, healthcare system, financial sustainability

## Abstract

Background: This study aimed to find out the application of a sustainability model framework to test the financial sustainability of the healthcare system in Saudi Arabia and to suggest some reforms required to maintain a sustainable healthcare system in the country.

Methods: To test the financial sustainability of the publicly funded healthcare system in Saudi Arabia, we applied analytical techniques using a sustainability model framework based on the framework indicators proposed previously by the Office of Sustainable Development, Bureau for Africa, U.S. Agency for International Development. An empirical time-trend analysis was also used to judge the financial sustainability of the healthcare system of Saudi Arabia in the future.

Results: The results showed significant threats to the financial sustainability of the healthcare system. Saudi Arabia's revenues, gross domestic product (GDP), government budget, and Ministry of Health (MOH) budget were all directly influenced by the oil prices.

Conclusion: The healthcare system in Saudi Arabia seems to be financially unsustainable, and the need for change is inevitable. Saudi's ambitious program of development “Vision 2030” will surmount the challenges faced by the country and will lead to substantial enhancements in the health sector in Saudi Arabia, and other opportunities for improvement do exist.

## Introduction

Sustainability is a vast notion, and it is difficult to define because it includes philosophy, ecology, economy, sociology, and many other disciplines. The most famous definition of the concept emerged from the World Commission on Environment and Development Report of 1987, which states that sustainability is “development that meets the needs of the present without compromising the ability of future generations to meet their own needs" [1]. Sustainability in healthcare systems remains one of the most disputed issues due to the economic expenses resulting from the provision of healthcare services and the increasing requirement for these services [2]. The sustainability of healthcare systems is a topic of growing importance, despite long debates. This importance resulted from the changing considerations of leading healthcare organizations and the interest in assessing their long-term capability. Financial sustainability, which can be defined as preserving a balance between revenue and expenses, is of paramount importance to the healthcare market, as it is the case in various types of businesses [3].

Although the concept of financial sustainability has been widely circulated through academic, media, and political platforms, this concept is not agreed upon and is dealt with in different forms. In addition, the discussion about healthcare systems rarely deals with how to obtain financial sustainability or what are the political aspects resulting from the problems of this sustainability [4]. The debate on the sustainability of healthcare systems focuses mainly on ways to finance these systems [5]. According to the World Health Organization, healthcare system financing can be defined as the process of accumulating income from primary and secondary sources, storing it in cash pools, and allocating it to healthcare providers [6]. In any health system, a major concern is about raising sufficient resources as “a good health financing system raises adequate funds for health, so that people can use needed services protected from financial catastrophe or impoverishment associated with having to pay for them. It provides incentives for providers and users to be efficient" [7].

Publicly financed healthcare systems across many countries are encountering a challenge in maintaining their financial sustainability as healthcare consumes a continually boosting portion of both the country's treasure and government expenditure. Resource scarcity, rising health spending as a proportion of the gross domestic product (GDP), and rises in the costs of health services due to factors affecting the demand and supply of these services are frequently quoted as grounds why healthcare systems need ever-increasing grants and reasons why inclusive public systems are not sustainable [4,5]. The report from the World Economic Forum in 2013 stated that the rise in healthcare spending is secondary to the aging population, the shift to chronic diseases secondary to lifestyle, increased public expectations, and lack of value-care principle among recipients of healthcare services [8].

Since the 1970s, Saudi Arabia has placed great emphasis on providing healthcare to its citizens. Over the past 50 years, enhancements have been made in the capacity and excellence of healthcare services provided in the Kingdom. The public sector, including the Ministry of Health (MOH) and other healthcare facilities funded by the government, and the private sector are the providers of healthcare services in Saudi Arabia. The MOH remains the major supplier of healthcare services in Saudi Arabia, and its services are for free. The MOH has funds derived from the annual total government budget obtained mostly from oil revenues [9-12]. The evident achievements of Saudi Arabia's healthcare system in the recent years can likely be credited to the high level of funding. This great endeavor to enhance healthcare services has been reflected in a substantial rise in the assigned budget, from 5.4% of the government’s total budget in 1990 to 7.25% in 2015. In 2019, the MOH budget accounts for 6.2% of the overall state budget and around 40% of the division of "Health and Social Development" in the country [13].

Unfortunately, the healthcare system in Saudi Arabia is under increasing pressure in spite of the sizable funds that the government can allot currently. The burden is secondary to the relevant challenges confronted by all healthcare systems that have public funds as mentioned earlier. These challenges include aging population, changing disease patterns into chronic diseases, increasing inactive daily routines, growing expenses, and increasing user anticipations [9,12,14]. Although the Saudi authorities are still able to allot the necessary funds to the health sector, the healthcare system is apparently under stress due to fast surges in expenditure and needs for high-value healthcare while resources remain limited. In the medium to long term, the current situation seems unsustainable, especially with ambiguities associated with the fluctuation in oil prices [9]. It is most likely that the main potential influencer in the delivery of holistic healthcare is the financial situation facing the healthcare system [2]. Therefore, it is necessary to take into consideration how healthcare provision can be financed, especially in light of the financial pressures that the healthcare system is facing at the present time.

This paper addresses the financial sustainability of the healthcare system in Saudi Arabia in terms of the financing of health expenditure and discusses the possible measures that might achieve that sustainability.

## Materials and methods

**Figure 1:**
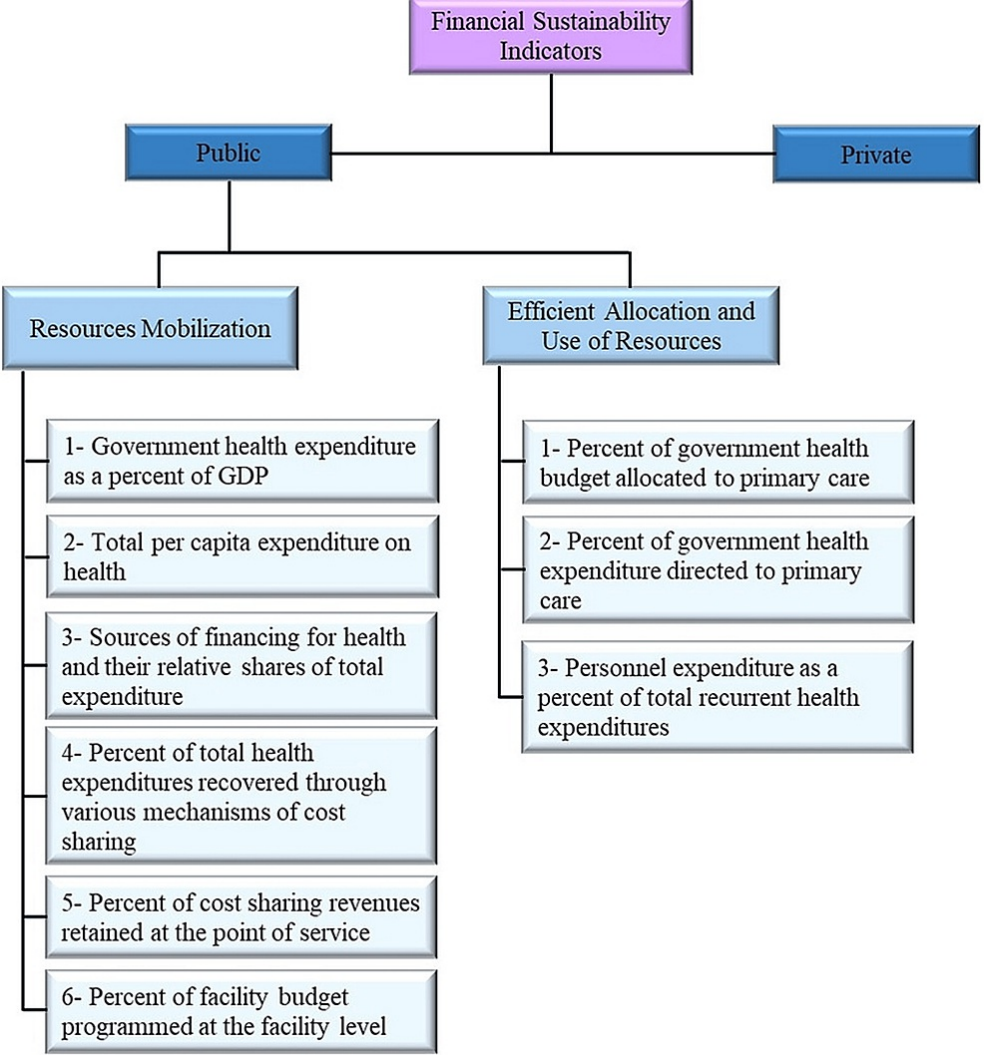
Sustainability model. Source: Office of Sustainable Development Bureau for Africa U.S. Vol. II Office of Sustainable Development (1999) [15]

To explore how the healthcare system is financed and to clarify the impact of a sustainability model on the financial aspect of Saudi Arabia's healthcare system, analytical techniques were utilized using a sustainability model framework. This sustainability model is based on the framework indicators proposed by the Office of Sustainable Development, Bureau for Africa, U.S. Agency for International Development in February 1999 (Figure 1).

The model is comprehensive and considers all the elements that contribute to sustainability. The indicators are measurement tools, used to investigate characteristics of ongoing healthcare systems and predict future sustainability. These indicators can be used for both process monitoring and evaluation [15].

We only employed the financial sustainability indicators grouped under the public sector financing category, as the primary goal is to investigate the financial sustainability of the healthcare system in Saudi Arabia, which is mainly supplied by a public sector. The model groups the financial indicators into two sub-elements: resource mobilization and efficient allocation and use of resources [15].

An empirical time-trend analysis was used in this study to judge the financial sustainability of the healthcare system of Saudi Arabia in the future. Public data sources were employed, and data used in this study have been collected from the official government websites of Saudi Arabia, including MOH statistical yearbooks, General Authority for Statistics annual yearbooks, Ministry of Finance budget reports, and Saudi Central Bank annual reports. 

Variables included in this study include Saudi Arabia's total revenue and expenditure (in millions), oil prices, percentage (%) of government budget, government budget (in millions), and total MOH budget (in millions) between 1990 and 2019. These variables have been chosen by this study as the key determinants of healthcare expenditure. Variables have been presented in their descriptive formats to check for their range, minimum, and maximum values.

## Results

**Figure 2:**
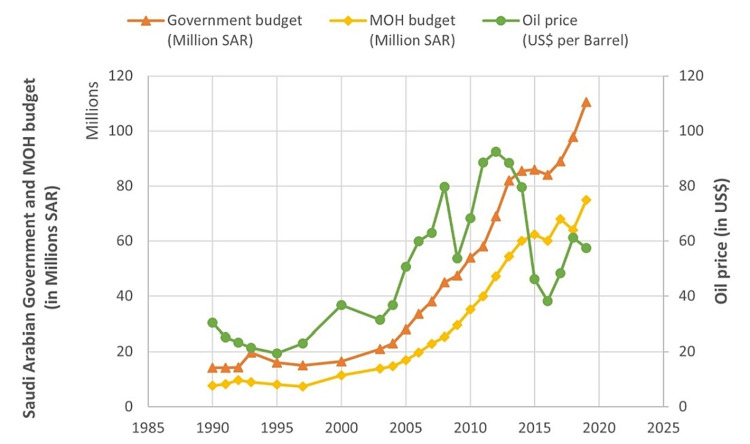
Oil price (in US$) and Saudi Arabian government and MOH budget (in millions SAR) (1990-2019). Source: MOH statistical yearbooks, General Authority for Statistics annual yearbooks, Ministry of Finance budget reports, and Saudi Central Bank annual reports

Looking at Saudi Arabia's economy between 1990 and 2019, we find that it relied heavily on oil prices, with the average participation of oil revenues calculated at 78% of the total revenues annually. It is also worth noting that the average participation of oil revenues from the total annual revenues has decreased over the past few years (2016 onward) to 65%, following the initiation of the impressive "Vision 2030." The heavy dependence on oil revenues led to many changes in economic activities in the Kingdom of Saudi Arabia during the mentioned years because of fluctuations in oil rates in the global market. Saudi Arabia's revenues, GDP, government budget, and MOH budget were all directly influenced by the oil prices. The relationship between oil prices, total revenues, and total expenditures is best seen where the steep upsurge in the oil prices in two periods, i.e., between 2003 and 2008 and between 2010 and 2013, resulted in a budgetary surplus, which had a positive impact on the government and MOH budget. The second-best example for the same relationship is seen in the period between 2014 and 2016, where the global crisis in oil markets led to the budgetary deficit and was reflected negatively on the government and MOH budget. The 1990s had an overall budgetary deficit that also led to a negative impact on the Saudi Arabian government and MOH budgets (Figure 2).

**Figure 3:**
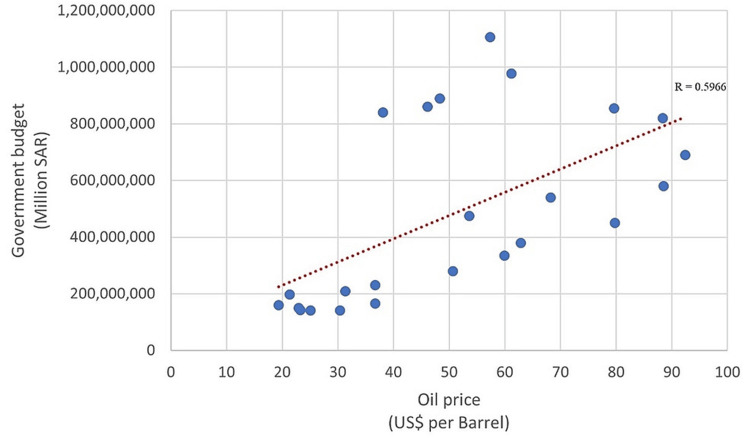
Correlation between the oil price (in US$) and Saudi Arabian government budget (in millions SAR) (1990-2019). Source: MOH statistical yearbooks, General Authority for Statistics annual yearbooks, Ministry of Finance budget reports, and Saudi Central Bank annual reports

It also worth mentioning that the Saudi Arabian government budget appears moderately correlated with oil prices (Figure 3).

**Figure 4:**
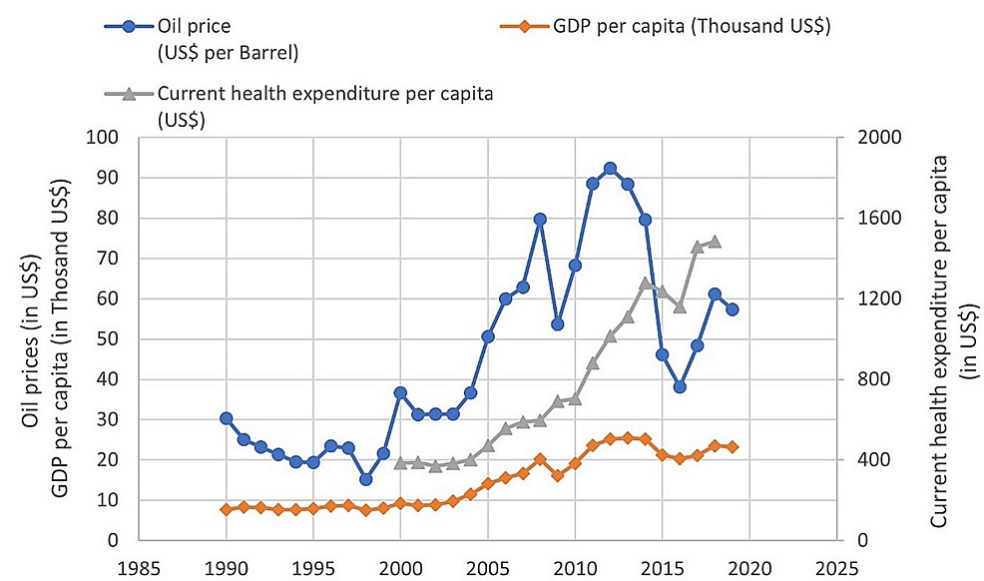
Current health expenditure per capita data are available only between 2000 and 2018 Source: MOH statistical yearbooks, General Authority for Statistics annual yearbooks, Ministry of Finance budget reports, and Saudi Central Bank annual reports

When taking an in-depth look at the budget of the MOH, the percentage of the MOH budget from the total state budget ranged between 4.15% in 1993 and 7.64% in 2017, and the average percentage of the MOH budget was 6.3% during the studied period. On the other hand, Saudi Arabia's total expenditures and current health expenditure per capita had a sharp rise that appeared a little affected by the fluctuation of oil revenues. Although Saudi Arabia's GDP has been affected by changes in oil prices, the current health expenditure per capita is gradually increasing with slight fluctuations, resulting from the change in the domestic national product (Figure 4).

**Figure 5:**
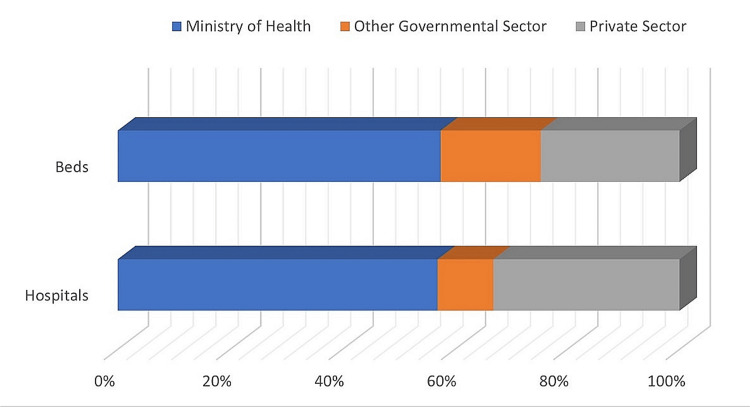
Healthcare sectors in Saudi Arabia and capacity. Source: MOH statistical yearbooks

The burden on the MOH can be easily demonstrated; MOH hospitals and bed capacity represent around two-thirds of the health service capacity in Saudi Arabia (Figure 5).

Based on the above-mentioned results, the proposed sustainability model was applied to test the financial sustainability of the healthcare system in Saudi Arabia and to highlight the areas of risk. Indicators affecting the financial sustainability of the public healthcare system as proposed in the analytical model were categorized into two key elements: resource mobilization and efficient allocation and use of resources. Most of the indicators showed unfavorable results. Table 1 lists the details of the results based on the indicators in the analytical model, in addition to a simplified explanation of each indicator.

**Table 1:** Sustainability model results

Key element	Indicator	Favorable/ unfavorable	Explanation
Resource mobilization	Government health expenditure as a percent of GDP	Unfavorable	Increasing the government health expenditure as a percent of GDP to adapt the natural increase of health expenditure and in the presence of the varying GDP secondary to the unpredictable oil prices may lead to system failure.
	Total per capita expenditure on health	Unfavorable	Steady rise in total per capita expenditure on health and the variations in the national income that correlates with the oil price instability indicate a higher sustainability risk.
	Sources of financing for health and their relative shares of total expenditure	Unfavorable	As the healthcare system is mainly publicly funded, the largest source of the funds arrives from the oil industry. Consequently, there is some sustainability risk if other sources are not successfully introduced.
Resource mobilization	Percent of total health expenditures recovered through various mechanisms of cost sharing	Unfavorable	Mainly affects the public sector, as there is no cost recovery. Full cost recovery in the private sector will increase the cost, limit its accessibility, and shift the burden to the public sector. This indicator may adversely impact the system in the absence of a well-organized and regulated private sector.
	Percent of cost-sharing revenues retained at the point of service	Unfavorable	The public sector has no retained cost-sharing revenue at the point of service. The risk involved with this indicator is similar to the previous indicator's risk.
	Percent of facility budget programmed at the facility level	Unfavorable	The budget is allocated at central level (MOH). Having this process in funding is against decentralization and ultimately a risk to the sustainability.
Efficient allocation and use of resources	Percent of government health budget allocated to primary care	---	This proportion cannot be calculated from the MOH budget data at this time, as the requisite data are not available (see the Discussion section).
	Percent of government health expenditure directed to primary care	---	This proportion cannot be calculated from the MOH budget data at this time, as the requisite data are not available (see the Discussion section).
	Personnel expenditure as a percent of the total current health expenditures	Unfavorable	Oftentimes, inefficiency is calculated by high staff expenditure and low drug and other services' spending. Employee compensation accounts for more than 60% of the MOH budget.

## Discussion

In this paper, having considered the funding of the healthcare system in Saudi Arabia and the challenges faced by the MOH, it is crucial to think about factors affecting the financial sustainability of the healthcare system and the essential measures to tackle those factors.

Unfortunately, there are no agreed-upon criteria for assessing and evaluating the financial sustainability of healthcare systems. Several groups of indicators have been proposed to assess the financial sustainability of healthcare systems. Generally, these indicators can be divided into two main groups. The first group is indicators based on evaluating health expenses and resources, while the second group is based on the attitudes of shareholders. When assessing the financial sustainability of any health system, the main indicators should be related to health spending and funds [16]. For a health service to be considered sustainable, the healthcare system operating the service must be able to produce and provide sufficient resources over the long term. In other words, the health service can be considered sustainable when there is a balance between capacity and activities over a certain period. This was the concept of another framework for the analysis of sustainability of healthcare systems proposed by the Development Partnership Center (DiS) in 1998 [17].

The proposed model framework utilized in this study has classified the indicators that test the financial sustainability risk of the system into two main key elements: resource mobilization and efficient allocation and use of resources [15]. It is worth mentioning that the same model has been used previously to assess the challenges faced by the healthcare system in a different country that shares many similarities with the system in Saudi Arabia [18]. Applying the proposed sustainability model showed that most of the financial indicators fall into the unfavorable domain. Hence, there is a sound financial sustainability risk, as highlighted by our results, and it appears to be multifactorial. Assessing the situation in the Saudi system revealed a correlation between the GDP and spending, where the driving force behind budget allocation is the GDP rather than demand. Despite the fluctuations in the GDP and the overall deficit, there was a tremendous increase in the per capita GDP. Accordingly, the percentage of GDP devoted to healthcare expenditure significantly rose. If the principle is increasing the government health expenditure as a percent of the GDP to adapt to the natural increase of health expenditure and in the presence of the varying GDP secondary to the unpredictable oil prices, applying this factor will indicate the major sustainability risk to the healthcare system in Saudi Arabia. Moreover, because higher healthcare spending in the GDP threatens the sustainability during fiscal recessions or downturns, it tends to jeopardize spending in other activities. This would lead to struggles concerning the country’s overall economic sustainability [19].

The WHO definition of healthcare system financing did not only include revenue collection but also fund pooling [6]. Revenue collection is mobilizing funds from different sources through multiple basic mechanisms that includes different types of insurance, different types of taxes, out-of-pocket payments, and transfers from donor agencies [20]. Meanwhile, fund pooling refers to gathering funds for the benefit or interests of all. Pooling basically indicates that contributors will share the financial risk [21]. Healthcare services in Saudi Arabia are mostly provided by the MOH, which is financed only by the government, predominantly from oil revenue. The reliance on a single contributor and resource for funding of the public sector appears to be another major threat to the financial sustainability of the system. Other resource mobilization risks that may endanger the financial sustainability of the system also included the lack of cost recovery through various mechanisms of cost-sharing and cost-sharing revenues retained at the point of service in the public sector and the centralized nature of budget allocation.

If assigning more resources to healthcare is aimed to attain fiscal balance as essential and an objective, then it may not make a difference how additional assets are created or spent. However, if the objective of assigning funds to healthcare is to participate in the fulfillment of health system targets, then the best strategy is to consider wisely how to utilize current resources and how to generate and use additional reserves [4]. Fortunately, the Saudi government disclosed an ambitious program of development in 2016 as “Vision 2030.” The program is a strategic plan by the Kingdom that aims to restructure the social and economic sectors of the country, including the healthcare system; it focuses on diversifying the country's economy and promoting several transformations in it.

Diversifying the source of funding for the health system would be the main solution for the financial sustainability risk and could be considered across many different platforms. Opening channels for funding networks should be achieved on two levels: at the national level and a local funding level. The national funding level means at the level of the central financing body, while the local funding level is at the level of the operator where the operator could generate some support to back-up the general funding shortage. Nevertheless, there is a necessity to enhance service funding and supply by developing collaboration and harmonization among different levels of the system and other shareholders.

**Figure 6:**
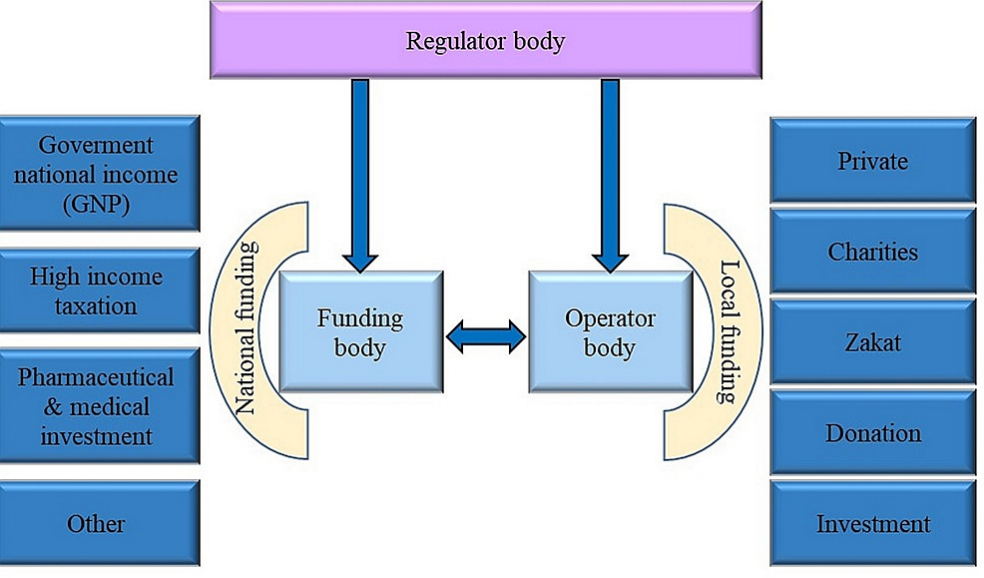
Suggested scheme for healthcare system organization and possible funding mechanisms at national and local levels. Suggested scheme by the author.

To achieve financial sustainability, the system must make strategic decisions and capital investments so that it can have satisfactory revenues to cover the healthcare needs for a certain period of time [3]. We have proposed a framework that can be used to control the generation of funding and its efficient allocation. The framework is shown in Figure 6.

The investment could be made through a funding agency and supported by the government. At the same time, revenue could be raised from the funding agency and not from national income. The regulatory body should oversee and regulate the research and development in this area. The Saudi Public Investment Fund (PIF) could be the potential funding agency, while the MOH can assume the role of the regulator body. The PIF as a global impactful investor with a focus on sustainable investments that can launch or continue strategic initiatives to grow the national economy; an example of such initiative is Saudi Arabia’s Economic Cities. It is assumed that this approach will produce additional revenue to the funding body's allocated budget and alleviate pressure on the GDP, and it will also promote public-private partnerships. The Kingdom’s ambitious blueprint "Vision 2030" also stated an important strategy: to make Saudi Arabia the investment powerhouse by significantly improving investment competitiveness and launching promising sectors in the Kingdom. The pharmaceutical and medical sectors are among the best industries to invest in, and they will support the healthcare system. Such industries may be used for self-reliant consumption and revenues.

Other sources of funding for the suggested framework could include “Zakat,” which is a mandatory religious taxation that has to be done annually. Saudis' attitude toward paying this tax is very acceptable, and people are happy to pay it. The allocating part of that tax for the funding of the healthcare system will provide additional resources, and the portion may be used in the frameworks of general and local funding. Moreover, voluntary personal donations could be seen as additional resources for the healthcare system. It could also be used at both the general and local levels of funding.

Furthermore, when considering the efficient allocation and use of resources, the proportion of government health budget allocated to and the spending on primary care is not evident from available data. However, the assumption for this part is that there is a tendency to assign unequal budgets and different expenditures between primary care and on the acute care or specialist levels, with the latter being preferred. This assumption, if confirmed, will indicate an inefficient resource allocation and utilization. The high staff cost and dependency on international labor both represent other unfavorable factors for efficient allocation and harbor a high sustainability risk.

Previous study investigating the National Health Service (NHS), the publicly funded healthcare system in England, linked the sustainability of the system to multiple factors. Effective health service provision and management of chronic illness is the primary aspect toward a sustainable healthcare system. Chronic diseases pose a risk to sustainability as they account for more than two-thirds of health expenses. Developing well-educated healthcare providers is another important contributor of financial sustainability [22]. With the launch of the health sector transformation plan as a part of “Vision 2030” to revamp the health sector's structure and funding, the government has reiterated its commitment to universal healthcare coverage. It also declared that an Essential Benefits Package (EBP) would be provided, and supplementary health insurance will be created. In addition, the plan depends on the principle of better value care, which ensures financial sustainability by strengthening public health and preventing diseases, thereby reducing avoidable illness and costs. The initiative also included plans to guarantee a proficient healthcare workforce, staffed mainly by Saudi citizens [23].

One of the main limitations in our study is that the fiscal space analysis has not been studied. Other factors affecting the sustainability of the system (e.g., inflation rates, aging population, and increase/decrease of the population) were not included in this paper. The framework proposed by Subhi Mehdi [15] did include all factors influencing sustainability. However, it has been demonstrated that detecting the essential factors to maintain the services is more effective [17]. Hence, only indicators relevant to the financial sustainability of the Saudi system were considered.

## Conclusions

With the current scenarios, the healthcare system in Saudi Arabia seems to be financially unsustainable as multiple financial indicators are unfavorable; therefore, the need for change is inevitable. The Kingdom of Saudi Arabia planned its “Vision 2030” to improve the economy and to surmount the challenges faced by the country. Our results support the emphasis to diversify the economy into a non-oil-revenue economy to promote economic growth and expansion of the Saudi nation. Although substantial enhancements have been made in the health sector in Saudi Arabia, other opportunities for improvement do exist. The rule of Saudi's Public Investment Fund or other governmental investment agencies would be a great opportunity for policymakers. Boosting the healthcare system funding could be achieved by enabling ways of recovering costs, sharing allocated budget by the local facilities, enhancing governance to achieve targets effectively and efficiently, and improving human resources for the health sector. In addition, the efficient use of resources, good management practice, and decentralization are crucial. Judicious amendments should be initiated in the health insurance industry to meet Vision 2030’s aims.
